# Effect of Dietary Enrichment with Flaxseed, Vitamin E and Selenium, and of Market Class on the Broiler Breast Meat—Part 1: Nutritional and Functional Traits

**DOI:** 10.3390/nu14081666

**Published:** 2022-04-16

**Authors:** Ambrogina Albergamo, Rossella Vadalà, Vincenzo Nava, Giovanni Bartolomeo, Rossana Rando, Nadia Colombo, Roberto Gualtieri, Massimiliano Petracci, Giuseppa Di Bella, Rosaria Costa, Nicola Cicero

**Affiliations:** 1 Department of Biomedical, Dental, Morphological and Functional Images Sciences (BIOMORF), University of Messina, Viale Annunziata, 98100 Messina, Italy; vnava@unime.it (V.N.); rrando@unime.it (R.R.); gdibella@unime.it (G.D.B.); costar@unime.it (R.C.); ncicero@unime.it (N.C.); 2 Science4Life Srl, an Academic Spin-Off of University of Messina, Viale Annunziata, 98100 Messina, Italy; gbartolomeo@unime.it; 3 Avimecc Spa, C.da Fargione, Agglomerato Industriale ASI, 97015 Modica, Italy; nadiacolombo@avimecc.com (N.C.); robertogualtieri@avimecc.com (R.G.); 4 Department of Agricultural and Food Sciences (DISTAL), Alma Mater Studiorum, University of Bologna, Piazza Goidanich 60, 47521 Cesena, Italy; m.petracci@unibo.it

**Keywords:** chicken meat, dietary enrichment, n-3 PUFAs, vitamin E, selenium, functional meat, FA composition analysis, element analysis

## Abstract

The effect of dietary enrichment with flaxseed, selenium and vitamin E, and market class on the nutritional and functional value of breast meat was evaluated. A completely randomized block design was set up, where the experimental unit (*n* = 6000 birds) received conventional or enriched diet and was slaughtered at 37 (light class), 47 (medium class), or 57 (heavy class) days of life. Hence, functional and standard *Pectoralis major* muscles from every market class were analyzed for FA composition, inorganic elements and vitamin E. Lipid metabolism indices and health lipid indicators were assessed along with the nutritional value. A multiple linear model revealed that in breasts, the dietary treatment significantly influenced (*p* < 0.05) the FA profile, lipid metabolism and health lipid indices, while the slaughtering weight was related (*p* < 0.05) to most of elements (e.g., Na, Mg, K, Mn, and Se) and vitamin E. The interdependence of the two factors had strong relations (*p* < 0.05) with total PUFAs, including linolenic acid, desaturase activities, health lipid indices, trace essential elements and vitamin E. Consequently, enriched meat from heavy chickens showed the best functional and nutritional traits. Overall, the study pointed out that both market class and dietary manipulation are two relevant factors to consider for producing breast meat with higher nutritional and functional value.

## 1. Introduction

In recent decades, the dramatic increase in consumer preference for chicken meat over beef, sheep and pork meat—related to evident cultural and economic issues, and a progressive adherence to healthier dietary patterns—and the noticeable shift in the poultry sector from an industry predominantly focused on whole bird commodity to a multi segment industry handling cut-up, deboned meat, and processed goods [1,2,3,4], have inevitably led to the increase in consumer expectations and the necessity for higher standards in chicken meat as well [2]. As a result, the broiler production system has been widely explored to ameliorate the quality of meat in terms of reducing muscle abnormalities [5,6,7] and improving technological [8], sensory [9], microbial [10,11] attributes, and, not least, nutritional/functional properties.

Considering the latter aspect, chicken meat has already demonstrated being an excellent matrix for designing functional food mainly because of the great versatility, which enables producers to obtain many attractive, convenient, and easy-to-use products, and the already significant presence of valuable and bioavailable nutrients (e.g., quality proteins, some essential fat-soluble vitamins, and minerals) [12,13]. The functionalization of poultry meat may occur either by dietary manipulation during the farming phase or by the enrichment with specific ingredients/molecules with functional/therapeutic properties during the meat processing [14,15,16,17].

The dietary manipulation in particular has widely pointed out how the guided intake of desirable healthy components tends to be reflected in the animal tissues, which gain advantageous biochemical, nutritional and functional traits [18,19,20]. On this basis, most effort has been devoted to ameliorating the fatty acid (FA) profile of chicken meat, especially in terms of n-3 polyunsaturated FAs (PUFAs), by the incorporation in diet of a variety of ingredients [21,22,23,24,25], including flaxseed [26]. Flaxseed is unique among seeds for the exceptionally high content of α-linolenic acid (ALA, up to 50%), but it is also source of anti-nutritional factors (i.e., mucilage, cyanogenic glycosides, trypsin inhibitor and phytic acid), which may negatively affect the health and well-being of the muscular mass of broiler, if not properly removed by the so called “extrusion” process [27]. In this respect, diets supplemented with extruded flaxseed demonstrated that the chicken safely absorbs most of the seed nutrients, including the essential ALA, which can be further metabolized into eicosapentaenoic acid (EPA) and docosahexaenoic acid (DHA) by desaturating and elongating enzymes [28,29].

The increased unsaturation degree is highly desirable for the positive effects on consumers’ health (i.e., activation of anti-inflammatory cascades, delay of the onset of aging-associated neurological degeneration, and reduction in the risk of certain cancers and cardiovascular diseases [30]. However, it may affect the oxidative stability of the meat, thus inevitably leading to the deterioration of its nutritional and functional value [31,32,33]. In such cases, the strategic combination of an n-3 PUFA source with antioxidant ingredients or molecules becomes mandatory for effectively slowing down the lipid oxidation, while enriching the meat with further functional nutrients [34,35]. Indeed, many studies already pointed out that a diet supplemented with extruded flaxseed and individual or combined antioxidants, such as vitamin E and selenium (Se), may positively influence the oxidative stability and healthiness of broiler meat [36,37,38,39,40].

In Italy, the system of broiler production usually provides the separate rearing of females and males to obtain three different market classes of birds, namely, light-, medium-, and heavy-size broilers. The light birds are 32–37-day-old females reared up to 1.2–1.7 Kg of live weight to produce 0.9–1.2 Kg carcasses intended for rotisserie products; medium birds are usually 42–49-day-old females (but may also be males) reared up to 2.3–2.8 Kg of live weight to yield 1.6–2.0 Kg carcasses mainly employed for cut-up products; heavy birds are 50–60-day-old male broilers reared up to 3.2–4.2 Kg of live weight to obtain 2.2–2.8 Kg carcasses for cut-up and further processed products [8].

To the best knowledge of the authors, the evolution of peculiar nutrients and functional traits of meat from broilers belonging to different market classes and receiving a functional or a conventional diet has not yet been investigated elsewhere. In fact, just two studies evaluated the effect of the slaughtering age and genetic strain on the FA profile and functional properties of meat from birds receiving a standard diet [41,42], whereas three papers investigated the influence of an enriched diet supplied to the chicken for an increasing time on the FA composition and functional potential of derived breasts [43,44,45]. However, both the dietary treatment and the market class represent factors that need to be further investigated, and also their interdependence, to better evaluate how certain metabolic changes can impact the meat quality of broiler chickens.

In light of this, the aim of the study was to investigate the influence of market class and dietary manipulation on breast muscles from broiler of different market classes. As the experimental diet was supplemented with extruded flaxseed meal (FSM), vitamin E and Se, the various products were investigated in terms of FA composition, element profile and vitamin E; insights on their functional and nutritional quality were also provided.

## 2. Materials and Methods

### 2.1. Experimental Design and Diets

A randomized complete block design was set on a total of 36,000 one-day-old broiler chicks (breed: Ross 308), which were farmed and slaughtered in the plant of the company “Avimecc S.p.A.” (Ragusa, Italy) during June–July 2021. The chicks were allocated into 72 flocks (500 birds/flock) designed to produce light (*n* = 24), medium (*n* = 24), and heavy (*n* = 24) broilers, and were housed from a minimum of 6 weeks to a maximum of 8 weeks, depending on the market class.

All flocks were randomly assigned to the experimental (i.e., enriched or functional) (*n* = 36, equally subdivided among the three market classes) and control (i.e., conventional) (*n* = 36, equally subdivided among the three market classes) feeding programs (Table 1). The birds were provided clean drinking water and were fed *ad libitum* with a starter diet during the first 10 days, a first grower diet up to day 24, a second grower diet up to day 39, and a finisher diet until the end of the experimental phase.

In both cases, the diets were iso-caloric and iso-nitrogenous to avoid confounding effects of variation in dietary energy and protein levels. In the case of the experimental diet, the amounts of supplemented ingredients were designed to meet or exceed the NRC requirements for broiler chickens [46]. The ingredients of both diets, as well as their proximate composition, are reported in Table 2.

At prefixed market ages, 5 representative broilers were randomly selected *per* flock, so that *n* = 120 birds from every market class, of which *n* = 60 fed the standard diet and *n* = 60 the experimental diet, could be slaughtered under commercial conditions (Table 1). In particular, light broilers were females slaughtered at 37 days of life, with a live weight ranging 1.7–2.0 kg; medium broilers were females slaughtered at 47 days of life, with a live weight of 2.5–2.8 kg; while heavy broilers were males slaughtered at 57 days of life, with a live weight of 3.2–3.5 kg. All the procedures were conducted according to the ethical norms of the Council Directive 2007/43/EC, laying down minimum rules for the protection of chickens kept for meat production [47].

### 2.2. Sample Collection

For each market class, boneless, skinless, *Pectoralis major* muscles coming from experimental (*n* = 60 split into three replicates of 20 samples each) and control (*n* = 60 split into three replicates of 20 samples each) birds were obtained at 3 h post-mortem in the deboning area of the plant. Subsequently, the breasts were packaged in zipper bags, tagged for identification, and immediately transported to the laboratory under refrigerated conditions. In the laboratory, every breast was trimmed of excess fat and connective tissue, and the obtained fillet was finely minced to obtain a homogenous meat mass, which was stored at −18 °C until further analysis. 

### 2.3. Chemicals and Reagents

Organic solvents, such as *n*-heptane and *n*-hexane (reagent grade) were purchased from J.T. Baker (Phillipsburg, NJ, USA), while *n*-hexane and ethyl acetate (HPLC grade) were supplied by LiChrosolv (Merk, Darmstadt, Germany). Fatty acid methyl esters (FAMEs) reference standards (C4–C24) and commercial standards of single tocopherols (α-tocopherol, β-tocopherol, γ-tocopherol and δ-tocopherol, 98% purity each) were from Supelco (Bellefonte, PA, USA). Trace metal analysis grade hydrogen peroxide (30% *v*/*v*) and nitric acid (65% *v*/*v*), and ultrapure water, with resistivity of 10 mΩ cm, were purchased from J.T. Baker (Milan, Italy). Stock solutions of Na, Mg, K, Fe, Cu, Mn, Zn, Se, As, Cd, and Pb (1000 mg/L in 2% HNO_3_, each) were provided by Fluka (Milan, Italy).

### 2.4. Fatty Acid Composition

The breast samples were elucidated for the FA profile according to a protocol of sample preparation and analysis already reported in Albergamo and colleagues [14]. For every sample (15.0 g), a Soxhlet extraction was performed with *n*-heptane for 6 h, and the lipid extract was evaporated to dryness with a rotating evaporator (Heidolph Instruments GmbH & Co., Schwabach, Germany).

After determining the extraction yield gravimetrically, the lipid extract was recovered by 1 mL *n*-hexane, and 10 drops of the mixture were added with 1 mL of sodium methoxylate and heated at 100 °C for 15 min. After cooling down the solution, 1 mL of boron trifluoride/methanol (14%) was added, and temperature was again raised to 100 °C for 15 min. Approximately 1 mL of *n*-hexane and 4 mL of a saturated sodium chloride solution were added to the cooled mixture. The organic layer with FAMEs formed after stirring was collected and analyzed by a gas chromatograph (GC) equipped with a split/splitless injector and a flame ionization detector (FID) (Dani Master GC1000, Dani Instrument, Milan, Italy). A Supelco Omegawax 250 (30 m × 0.25 mm I.D., 0.25 µm film thickness, Supelco, St. Louis, MO, USA) was employed. The following operating conditions were used: column oven temperature from 50 (hold time 2 min) to 240 °C (hold time 15 min) at 3 °C/min; injector and detector temperatures were both at 240 °C; helium (He) was at a linear velocity of 30 cm/s (constant), and an initial head pressure of 99.5 Kpa was set. The injection volume was 1 µL, with a split ratio of 1:50. Data acquisition and management were performed using Clarity Chromatography Software v4.0.2 (DataApex, Prague, Czech Republic). The identification of FAMEs of nutritional interest occurred by direct comparison with the retention times of reference FAMES, while the quantification was performed by considering the individual FAMEs (%) in relation to the total chromatogram area. Triplicate measurements along with analytical blanks were carried out for every sample.

#### 2.4.1. Fatty Acid Metabolism Indices

The FA metabolism of control and functional breasts was investigated by considering the activities of the thioesterase and elongase enzymes, which were measured in terms of conversion of myristic acid (C14:0) into palmitic acid (C16:0) and further into steric acid (C18:0) [48].

$$\mathrm{Thioesterase}\;\mathrm{index}=\frac{C16:0}{C14:0}$$

$$\mathrm{Elongase}\;\mathrm{index}=\frac{C18:0}{C16:0}$$

Desaturating enzymes, converting C16 and C18 saturated Fas (SFAs) into the respective monounsaturated Fas (MUFAs), were calculated in terms of two different Δ^9^-desaturase indices (DI), each resulting from relating the amount of the specific substrate to the corresponding product, and total Δ^9^-DI, as follows [28]:

$$\Delta^{9}{-\;\mathrm{DI}}_{C16}=100\frac{C16:1n-7}{C16:1n-7+C16:0}$$

$$\Delta^{9}{-\mathrm{DI}}_{C18}=100\frac{C18:1n-9}{C18:1n-9+C18:0}$$

$${\mathrm{Total}\;\Delta }^{9}-\mathrm{DI}=100\frac{C16:1n-7+C18:1n-9}{C16:1n-7+C16:0+C18:1n-9+C18:0}$$

Additionally, the efficiency of converting essential Fas, such as linoleic acid (LA) and ALA, into long chain PUFAs was calculated in terms of Δ^5^ + Δ^6^−DI [28]:

$$\begin{array}{l} \Delta^{5}+\Delta^{6}-\mathrm{DI}\\ =100\frac{C20:2n-6+C20:4n-6+\mathrm{EPA}+\mathrm{DHA}}{\mathrm{LA}+\mathrm{ALA}+C20:2n-6+C20:4n-6+\mathrm{EPA}+\mathrm{DHA}} \end{array}$$

#### 2.4.2. Functional Quality

The functional quality of fat from the different breast samples was determined by several health indices derived from the relative from FA profiles, such as the saturation index (S/P), the hypocholesterolemic and hypercholesterolemic FA ratio (h/H), the atherogenicity index (AI), and the thrombogenicity index (TI), as already reported by Albergamo and colleagues [49]. They were calculated as follows:

$$S/P=\frac{C14:0+C16:0+C18:0}{\Sigma \mathrm{MUFA}+\Sigma \mathrm{PUFA}}$$

$$h/H=\frac{C18:1n-9+\;C18:2\;n-6+\;C18:3n-3}{C14:0+\;C16:0}$$

$$\mathrm{AI}=\frac{C14:0+4\left(C16:0\right)}{\Sigma \mathrm{MUFA}+\Sigma n-3+\Sigma n-6}$$

$$\mathrm{TI}=\frac{C14:0+C16:0+C18:0}{0.5\left(\Sigma \mathrm{MUFA}\right)+3\left(\Sigma n-3\right)+0.5\left(\Sigma n-6\right)+3\left(\Sigma n-3/\Sigma n-6\right)}$$

Additionally, another important determinant of health, namely the ratio of n-6/n-3 Fas, was evaluated [50].

### 2.5. Profile of Inorganic Elements

For the screening of inorganic elements in breast samples, every sample (0.5 g) was added with 7 mL of HNO_3_ and 1 mL of H_2_O_2_, and subsequently mineralized through a microwave digestion system (Ethos 1, Milestone, Bergamo, Italy), by raising the temperature from 0–200 °C in 10 min (hold time 10 min), with a microwave power of 1200 W. After a proper dilution in ultrapure water, the digested sample was analyzed by a quadrupole ICPMS iCAP Q (Thermo Scientific, Waltham, MA, USA), according to a method of analysis previously optimized to reduce spectral (polyatomic and isobaric) and non-spectral interferences, according to what already described in our previous works [51,52,53,54,55,56]. The screening of minerals (i.e., Na, Mg and K), trace essential elements (i.e., Mn, Fe, Cu, Zn and Se), and potentially toxic elements (i.e., As, Cd and Pb) was performed according to a procedure reported elsewhere [57,58,59]. The instrumental conditions were: incident radio frequency power 1500 W; plasma gas flow rate [argon (Ar)] 14 L/min; auxiliary gas flow rate (Ar) 0.9 L/min; carrier gas flow rate (Ar) 1.10 L/min, collision cell gas flow rate (He) 4.0 mL/min. The spray chamber was set at 2.0 °C, while the injection volume and the sample introduction flow rate were set to 200 µL and 1 mL/min, respectively. Spectra acquisition occurred in full scan mode (dwell time 0.5 or 0.01 s/point, depending on the analyte). Data acquisition and processing occurred through Qtegra™ Intelligent Scientific Data Solution (Thermo Scientific™). For quantification, suitable six-point calibration curves were constructed for each analyte. Triplicate measurements along with analytical blanks were performed for every sample. 

### 2.6. Vitamin E

The determination of vitamin E, intended as sum of α-, β-, γ-, and δ-tocopherol, occurred according to a protocol of sample pretreatment and analysis proposed by Rotondo and coworkers [60], with slight modifications. For every sample, 0.1 g of the lipid extract obtained as described in Section 2.2 was put into a dark screw-cup vessel, dissolved in 1 mL of *n*-hexane, and further added with 1 mL of methanolic KOH. The mixture was first sonicated for 10 min at 20 °C and then centrifuged (4000× *g*, 10 min, +4 °C), to obtain a supernatant which was filtered through a 0.20-μm pore syringe filter, transferred into an amber vial, and analyzed by high-performance liquid chromatography coupled to fluorescence detector (HPLC/FD). Analyses were performed by an HPLC system (Shimadzu, Milan, Italy) consisting of a CBM-20A controller, a CTO-20A column oven, an LC-20AD pump, a DGU-20A3 degasser, and an RF-20A fluorescence detector. A LiChrosorb^®^ Si60 column (250 mm × 4.6 mm I.D., 5 µm particle size, Merck, Darmstadt, Germany), protected by a LiChroCART 4-4 guard column with the same stationary phase (Merck, Darmstadt, Germany), was employed. Analyses were performed at 40 °C, under isocratic conditions, with a mobile phase consisting of *n*-hexane/ethyl acetate (90:10 *v*/*v*). The injection volume was 20 µL and the flow rate was 0.8 mL/min. Data were processed by means of LabSolutions software ver. 5.10.153 (Shimadzu). The identification of tocopherols was carried out by a direct comparison with the retention time of relative commercial standards at excitation and emission wavelengths of 295 nm and 330 nm, respectively, while the quantitative analysis was performed by constructing appropriate calibration curves for every tocopherol, and by calculating the content of vitamin E as the sum of single compounds. Triplicate measurements along with analytical blanks were conducted for every sample.

### 2.7. Statistical Analysis

The number of flocks intended for functional (*n* = 12), or standard (*n* = 12) feeding was considered as the experimental unit. As a result, analytical data were expressed as mean ± standard deviation of triplicate measurements *per* experimental unit.

Once experimental data were submitted to a Shapiro–Wilk test to meet the initial assumptions, they were analyzed by the GLM procedure of the SAS statistical package (SAS Institute Inc., Cary, NC, USA), according to a multiple linear model that included the concentrations of analytes in breast meat as dependent variables, and the dietary treatment and the market class as the fixed factors. The model was described by the equation:

$$y=\beta_{0}+\beta_{1}x_{1}+\beta_{2}x_{2}+\beta_{1,2}x_{1}*x_{2}$$

where *y* is the predicted value of the dependent variable, *β*_0_ is the y-intercept, *β*_1_x_1_ is the regression coefficient of the first independent variable (i.e., market class), *β*_2_x_2_ is the regression coefficient of the second independent variable (i.e., dietary treatment), and *β*_1_,_2_$x_{1}*x$_2_ is the regression coefficient of the interaction between the independent variables. The statement of significance was set a *p* < 0.05.

In case of significant relation of the analytes with the market class of broilers, significant differences among the mean values were established by a post hoc Tukey’s honestly significant difference (HSD) test. Statistical significance was accepted at *p* < 0.05.

## 3. Results and Discussion

### 3.1. FA Profile

The total lipid content and the FA composition of control and functional samples from light, medium and heavy broilers are reported in Table 3.

Total fat significantly increased in breast meat as the market age at slaughter of broiler increased (1.68–4.03%, *p* < 0.05), while significantly reduced in functional samples with respect to the conventional ones (3.13% vs. 2.62%, *p* < 0.05) (Table 3). Additionally, the statistical model revealed a significant relation between the market class × dietary treatment interaction (*p* < 0.05), thus indicating that the fat tended to increase to a lesser degree in breast muscles of heavier chickens receiving the experimental diet than the standard ones. These findings are in line both with those studies reporting a physiological increase in intramuscular fat of pectoral muscles as the slaughtering weight of broiler increased [61,62] and highlighting a distinctive reduction in intramuscular fat as direct consequence of the FSM supplementation in the diet [28,45,63].

In breast muscles, the proportion of SFAs was not affected by the market class of broilers (36.48–34.35%, *p* > 0.05), but the enrichment of diet led to a consistent lowering of this FA class (from 37.78% to 33.39%, *p* < 0.05). Additionally, a significant interaction between the two factors pointed out that total SFAs would decrease in heavier broilers receiving functional feed. The predominant SFA, i.e., palmitic acid, has clearly affected the behavior of total SFAs in breasts. Indeed, it slightly lowered in meat with the increasing slaughtering weight of broiler (26.91–25.45%, *p* > 0.05), being in similar contents in samples from light and medium specimens, and significantly reduced going from standard to enriched breast muscles (28.33% vs. 25.25%, *p* < 0.05). Additionally, the model showed a significant relationship between these FAs and the market class × treatment interaction (*p* < 0.05) (Table 3).

The trend of predominant SFAs observed over the life span of birds was also reported in the breast meat from broilers fed standard diets and slaughtered at 9 and 18 weeks [42], and at 81 and 70 days of life [41], as well as in the breast muscle from chickens fed a diet enriched with FSM for an increasing duration of time [43,45]. On the other hand, the supplementation of FSM in diet already proved to reduce the content of most abundant SFAs by reducing the expression of enzymes involved in the lipogenesis process, such as the acetyl-CoA-carboxylase and the fatty acid synthase [64,65], as well as by enhancing elongation and desaturation activities, which promote the conversion of SFAs into longer PUFAs [43].

Neither market class nor dietary enrichment, nor their interaction, were significantly related to single and, thus, total MUFAs in the breast meat (*p* > 0.05). Oleic acid, for example, ranged between 31.30–32.40% (*p* > 0.05) among light medium and heavy broilers, and control and experimental breast muscles showed basically similar levels of such FAs (31.40–31.70%, *p* > 0.05) (Table 3). Accordingly, a univocal trend of single MUFAs in broiler meat in relation to the slaughtering weight or the dietary treatment cannot be inferred from literature. In fact, breast meat from broilers supplied with standard diets and slaughtered at increasing age reported a general reduction in both palmitoleic and oleic acids [41,42]. On the other hand, breasts from broilers fed a flaxseed-supplemented diet for different duration were marked by and increasing content of both palmitoleic and oleic acid [45], as well as by higher palmitic and lower oleic acid [43].

The total PUFAs of breasts showed significant linear relations (*p* < 0.05) with the market class, the dietary treatment, and not least the market class × dietary treatment interaction. In fact, PUFAs increased when moving from lighter to heavier chickens (26.90–27.44%, *p* > 0.05) and following the diet functionalization (24.18% vs. 29.60%, *p* < 0.05), thus resulting in the highest levels in enriched breasts from heavy birds. Although not predominant, ALA was the only FA to reflect the same behavior of total PUFAs, thus being the n-3 PUFA most affected by both the market class and the manipulation of diet with extruded FSM. ALA, in fact, became higher in the various samples as the slaughtering weight of broilers increased (4.90–6.54%, *p* > 0.05) and was found at significantly higher levels in enriched than standard products (2.77% vs. 8.18%, *p* < 0.05). The other n-3 PUFAs, namely, EPA and DHA, were not significantly related to the market class (*p* > 0.05), probably because of similar levels of such FAs in products from light and medium birds (EPA: 0.30–0.44%, *p* > 0.05; DHA: 0.44–0.50%, *p* > 0.05). However, a significant relation was established with the dietary treatment (*p* < 0.05), since both FAs resulted significantly increased in meat from broilers fed FSM-supplemented diet (EPA: 0.11% vs. 0.58%, *p* < 0.05; DHA: 0.23% vs. 0.69%, *p* < 0.05). Considering n-6 PUFAs, LA was neither correlated with market class nor with dietary treatment, although it clearly decreased from standard to enriched products (20.08% vs. 18.06%, *p* > 0.05) However, eicosadienoic acid and ARA had a significant relationship with the dietary treatment, and, equally to LA, they reduced in functional meats (eicosadienoic acid: 0.38% vs. 0.25%, *p* < 0.05; ARA: 0.89% vs. 0.49%, *p* < 0.05) (Table 3). 

Concerning the broiler age at slaughter, our findings were in line with previous studies reporting higher levels of EPA and DHA in the breast of chickens slaughtered at 18 weeks of age than 9 weeks [42], at 21–42 days of life than 7–21 days [66], at 81 days of life than 70 days [41], as well as broilers fed FSM for increasing time periods [45]. The higher levels of long-chain PUFAs in muscles of heavy birds may be related to an increased activity of enzymes involved in their synthesis, such as Δ^5^- and Δ^6^-desaturases and elongases, with advancing age [67]. 

Considering the dietary treatment, two previous studies agreed to reveal in enriched breasts not only higher PUFAs at the expense of SFAs and MUFAs [28,43], but also n-6 FAs reduced in favor of n-3 FAs [28]. Lower values of n-6 FAs could be the consequence of competition between n-3 and n-6 FAs for Δ5- and Δ6- desaturases involved in the biosynthesis of PUFAs such as AA, EPA, and DHA [68]. Moreover, in accordance with our findings, Poureslami and colleagues [66] pointed out significant relations of ALA with diet, age, as well as diet×age interaction in chicken whole body meat, while Mirshekar and coworkers [43] highlighted in chicken breast a strong positive linear relationship between n-3 FAs and the duration of flaxseed consumption, thus, indicating that the longer the consumption of flaxseed, the higher the n-3 content in the derived meat.

The increase in total PUFAs and n-3 FAs is a well-known effect of feeding programs based on flaxseed alone or in combination with other antioxidant components [28,36,38,39,45,69,70,71]. In particular, the supplementation of antioxidants, including vitamin E and selenium, would protect the increased n-3 Fas of meat from oxidation [72,73,74]. A higher level of EPA and DHA is one of the most desired effects of meat functionalization, since humans show poor ability to convert ALA into EPA and DHA, due not only to the competition between n-3 and n-6 essential Fas for desaturation and elongation activities [75], but also to the greater ALA oxidation with the increase in its intake [76].

#### 3.1.1. Fatty Acid Metabolism 

The FA metabolism indices derived from the FA profiles of standard and enriched pectoral muscles from light, medium and heavy chickens are reported in Table 4.

In the fatty acid synthase complex, thioesterase is responsible for terminating the cycle of FA synthesis and releasing the newly synthesized FA. As both the C14-acyl acyl carrier protein (ACP) and the C16-acyl ACP are the substrates of such enzyme, and the palmitic acid its main product, the ratio of C16:0 to C14:0 was exploited to reflect the selective cleavage of thioesterase on C14-acyl ACP. Hence, the greater the thioesterase index, the lower the cleavage of C14:0 and the higher the C16:0 production. On the other hand, the elongase index was calculated as the ratio of C18:0 to C16:0, and it is indicative of the extent of the subsequent synthesis of C18 FAs [77].

The thioesterase index was strongly related only to the market of class of broiler (*p* < 0.05). Accordingly, such index progressively increased in breast meat as the weight of birds increased (32.12–43.27, *p* < 0.05) (Table 4); however, this was due to the gradual reduction in palmitic acid already observed in the FA composition of relative samples (Table 3). Conversely, the elongase index was constant regardless of the market class (0.32, Table 4), this finding corresponding to a quite constant level of stearic acid in the FA profile of investigated samples (Table 3). The enrichment of diet did not establish a significant relationship with both indices, which were quite similar in meat from experimental and control samples (thioesterase: 38.53 vs. 36.73, *p* > 0.05; elongase: 0.30 vs. 0.34, *p* > 0.05) (Table 4).

The same evolution of thioesterase index in relation to the slaughtering age was observed in the breast of chickens slaughtered at 18 weeks of age compared to 9 weeks [42], as well as fed FSM from 1 to 5 weeks of age [45]. Nevertheless, the literature reached conflicting conclusions about the elongase index, as on one hand the age of broilers positively influenced the activity of the enzyme [42,66], while on the other hand a diet supplemented with FSM and supplied to broilers for an increasing duration did not affect it in the breast meat [45]. Contrasting evidence about the influence of diet on the two indices was also described, as Kumar and colleagues highlighted an increasing activity of thioesterase related to the addition of FSM in the diet of broilers, alone and in combination with turmeric, while no diet effect was observed for the elongase index [28,45].

The Δ^9^-desaturases catalyze the conversion of endogenous and dietary palmitic and stearic acid into the corresponding palmitoleic and oleic acid, and they can be intended as an indirect index of the stearoyl-CoA desaturase activity [77]. According to the obtained data, Δ^9^-DI_C16_ and Δ^9^-DI_C18_ and total Δ^9^-DI were not significantly related to the market class (*p* > 0.05), probably because of similar enzymatic activities in products from light and medium birds, and become higher only in the samples from heavy specimens (Δ^9^-DI_C16_: from 11.98–12.11 to 13.75, *p* > 0.05; Δ^9^-DI_C18_: from 78.52–78.66 to 79.80, *p* > 0.05; total Δ^9^-DI: from 39.45–39.91 to 41.55, *p* > 0.05). Overall, these data corresponded to the slight increase in palmitoleic and oleic acids observed in the FA composition of breasts (Table 3). With respect to the feed manipulation, the desaturase activities were higher in functional than conventional breast muscles (Δ^9^-DI_C16_: 13.09 vs. 12.07, *p* > 0.05; Δ^9^-DI_C18_: 78.57 vs. 79.42, *p* < 0.05; total Δ^9^-DI: 38.65 vs. 41.96, *p* > 0.05). However, only Δ^9^-DI_C18_ showed a significant linear relation with the dietary treatment, and only the total Δ^9^-DI had a relevant relationship with the market class × dietary treatment interaction. In this respect, the shift in the FA metabolism towards the synthesis of precursors of longer PUFAs, at the expense of saturated and shorter FAs, would enhance with the increasing age of broiler, as well as with the feeding manipulation (Table 4).

The literature reported contrasting findings on the linear relationship between the activity of Δ^9^-desaturase and the age of broilers. Different authors, for example, stressed that such enzymatic activity tended to increase as the age of the broiler increased, both under conventional and FSM-supplemented feed [45,66,67]. Conversely, Popova and coworkers discovered that the content of MUFAs in the breast decreased with age, as a reflection of a decreased Δ^9^-desaturase activity [42]. Concerning the dietary treatment, Kumar and colleagues confirmed the increase in every desaturase in breasts of chickens fed FSM (alone or in combination with turmeric) with respect to those obtained by conventional feed [28,45]. In the case of a diet supplemented with FSM and turmeric, theΔ^9^-DI_C18_ in particular became higher with the increase in the antioxidant source to FSM ratio in the diet, because of the protective role played by the antioxidants on the FA metabolism [28].

Although animals cannot synthesize LA and ALA from acetyl-CoA, they can convert such Fas into more unsaturated and longer-chain FAs, by means of desaturating, elongating, and terminating enzymes. Hence, the Δ^5^ + Δ^6^-DI was employed in this study to calculate the ability of broilers to synthesize long-chain FAs, such as eicosadienoic acid, AA, EPA, and DHA, from C18 Fas such as LA and ALA [77]. Similarly to the Δ^9^ desaturases, Δ^5^ + Δ^6^-DI was not related to the market class of broilers, although a slight and non-significant increase was observed in breasts from lighter to heavier classes (6.10–7.50, *p* > 0.05). However, the dietary enrichment had a strong relationship with the index, which significantly increased in the enriched products with respect to the standard ones (5.03 vs. 8.44, *p* < 0.05). Moreover, according to the statistical model, the market class × dietary treatment interaction was positively related to the activity of these enzyme (*p* < 0.05), which consequently would be more pronounced in functional products from heavy specimens (Table 4).

In accordance with our results, broilers fed FSM from 1 to 5 weeks of age showed a progressive increase of Δ^5^ + Δ^6^-DI [45]. However, Poureslami and colleagues reported a decrease in the apparent desaturation activity in relation to the age, as it was significantly higher in whole body meat from broilers slaughtered at 7–21 days of age than at 21–42 days of age [66]. When considering the feed manipulation, the literature pointed out a boosted activity of Δ^5^ + Δ^6^ desaturases in the breast muscles of birds fed FSM (alone or in combination with turmeric powder) than with conventional diet [28,45].

#### 3.1.2. Functional Traits

The functional indices derived from the FA profiles of standard and enriched pectoral muscles from light, medium and heavy broilers are listed in Table 5.

The S/P index is a generic index of the degree of saturation of lipids in the food matrix, which assumes that all SFAs and PUFAs are equivalent to each other [78], whereas the h/H ratio is indicative of the effect of individual Fas on the consumer’ s cholesterol metabolism. As a rule, the higher the h/H ratio, the greater the PUFA content and thus the benefits for human health [79]. Overall, S/P and h/H were not significantly related to the market class (*p* > 0.05), probably because of similar indicators in products from light and medium birds, which varied only in samples from heavy specimens (S/P: from 0.59 to 0.54, *p* > 0.05; h/H: from 2.04–2.06 to 2.19, *p* > 0.05). On the other hand, a strong linear relationship was found between these indices and the feeding manipulation (*p* < 0.05). Accordingly, S/P decreased and h/H increased in enriched breasts when compared to the standard counterpart (S/P: 0.63 vs. 0.52, *p* < 0.05; h/H:1.87 vs. 2.33, *p* < 0.05) (Table 5). These findings are a clear consequence of the FSM-enriched diet, which led to the decrease in SFAs in favor of unsaturated FAs (Table 3), due to the enhancement of the Δ^9^_c18_, Δ^5^ and Δ^6^ desaturase activities already observed in this study (Table 4) and elsewhere [28,45,67]. 

The AI characterizes the atherogenic power of food, i.e., its probability to cause atherosclerosis, in relation to the proportion of pro-atherogenic FAs (i.e., C14:0, and C16:0) and anti-atherogenic FAs (i.e., MUFAs, as well as n-3 and n-6 FAs) in the FA composition, while the TI defines the thrombogenic potential of the food matrix, i.e., its propensity to induce thrombosis, depending on the relative contribution of pro-thrombogenic FAs (i.e., C14:0, and C16:0) and anti-thrombogenic FAs (i.e., oleic acid, LA, and ALA) to the FA profile [80]. Another relevant indicator, namely, the n-6/n-3 ratio, was exploited for explaining the influence of dietary PUFAs on the pathogenesis of cardiovascular diseases, cancer, inflammatory and autoimmune disorders as well. In the most simplistic interpretation, an n-6/n-3 ratio close to 10 is considered detrimental for human health, while a value as close as possible to 1 is considered protective against such pathologies [50].

According to the results, the AI, TI and n-6/n-3 ratio did not show a clear relationship with the market class (*p* > 0.05), as they were similar in products from light and medium birds and significantly reduced in those from the heavy ones (AI: from 1.77–1.75 to 1.62, *p* > 0.05; TI: from 0.85–0.84 to 0.69, and n-6/n-3: from 5.83–5.42 to 3.25, *p* > 0.05). Nevertheless, the three indices were remarkably related to the dietary regime, as the redesign of diet induced their significant decrease in the functional samples when compared to the conventional counterpart (AI: 1.91 vs. 1.51, *p* < 0.05; TI: 1.00 vs. 0.58, *p* < 0.05; n-6/n-3: 7.52 vs.2.15, *p* < 0.05) (Table 5). In line with our results, the literature reported a decrease in AI, TI and n-6/n-3 in breast muscles from broilers fed FSM (alone or in combination with turmeric powder) than products from chickens fed a standard diet [28,45].

To the best knowledge of the authors, there are no previous reports on the evolution of functional indices of breast meat over the life span of the broiler. Only one previous work investigated the same indicators in breast muscles of broilers slaughtered at 70 and 81 days of age, albeit without finding a precise trend [77]. Although the multiple linear model did not reveal a statistically significant (*p* > 0.05) influence of market class (Table 5), every functional indicator was characterized by a strong market class × dietary treatment interaction (*p* < 0.05), which would explain the ameliorated functional traits of enriched products from heavy broilers. In particular, the improvement in functionality of the breast meat from heavy birds can be expected, since the latter physiologically showed lower saturation and higher unsaturation degrees in muscles (Table 3). 

Overall, the improvement of the functional traits in heavier chickens, as well as through feeding manipulation, led to the production of a healthier breast meat, whose regular consumption may prevent the occurrence of cardiovascular diseases and other disorders related to dyslipidemia. In particular, based on the calculated n-6/n-3 ratios, the consumption of products from heavier birds (ratio close to 4:1) would already protect from cardiovascular diseases while the functional meat (ratio < 2.5:1) would also reduce the risk of colorectal cancer, typically associated with the high consumption of meat [50].

### 3.2. Inorganic Elements

The element fingerprint of standard and experimental breasts from broilers belonging to the different commercial classes is reported in Table 6.

Minerals, such as Na, Mg and K, established a significant relationship with the market class (*p* < 0.05). However, they were generally comparable in breasts from light and medium birds and significantly higher (*p* < 0.05) in the heavy ones (Na: from 14.93–15.88 mg/100g to 27.44 mg/100g; Mg: from 245.10–250.01 mg/100g to 328.46 mg/100g; and K: from 424.29–411.27 mg/100g to 473.36, *p* > 0.05). Although such minerals were sensibly higher in functional than conventional meats (Na: 6.52 mg/100g vs.32.32 mg/100g, Mg: 66.84 mg/100g vs. 482.20 mg/100g; K: 40.21 mg/100g vs. 799.06 mg/100g), they did not significantly relate to the supplementation of Se in diet. However, Mg had a strong relationship with the market class × dietary treatment interaction, thus resulting in positively affected enriched breasts from heavy birds (Table 6).

To the best knowledge of the authors, no study has ever been conducted on the content of minerals in broilers from different market classes, nor following functionalization of the diet with Se. However, in line with the significant relation mineral-market class, the increase in minerals displayed in products from light to heavy birds could be related to the increasing requirement of minerals during the growth phase, these elements being involved in many metabolic processes [81,82]. On the other hand, in functional meats, the higher levels of these metals would be not related to the supplementation of Se in the diet. Indeed, Yao and colleagues demonstrated that following Se deficiency treatment, the contents of these macro elements were not significantly influenced in the breast muscle [83]. Consequently, the greater levels of such minerals could be explained by their reduced dietary supply through the premix (see Table 2) in favor of Se, which is usually counterbalanced by an enhanced tissue storage and a reduced excretion [82]. Additionally, the remarkable increase in Na in meat could be related to the intake of inorganic Se in the form of Na_2_SeO_3_ (see Table 2).

Trace essential elements, such as Fe, Zn, Mn and Se, became progressively higher in breasts from light to heavy chickens (Fe: 1.36–2.21 mg/100g; Zn: 0.27–0.40 mg/100g; Mn: 22.48–31.19 µg/100g, and Se: 8.35–20.11 µg/100g, *p* > 0.05). Similarly, the experimental products reported a uniform increase in all these elements with respect to the conventional counterparts (Fe: 0.62 mg/100g vs. 2.75 mg/100g; Zn: 0.31 mg/100g vs. 0.54 mg/100g; Mn: 6.33 µg/100g vs. 48.06 µg/100g, *p* < 0.05; Se: 5.33 µg/100g to 20.53 µg/100g *p* < 0.05) (Table 6).

The multiple linear model revealed peculiar relations for every element. Fe and Zn, for example, had significant relations with the dietary treatment and the market class × dietary treatment interaction (*p* < 0.05), respectively, whereas Mn was significantly related to both the commercial class of broilers and feed manipulation (*p* < 0.05). Interestingly, Se was characterized by relevant interactions with all the sources of variation investigated, thus resulting in a greater extent of retention in the muscle tissues of heavier birds receiving a Se-supplemented diet (Table 6).

The age of broiler already demonstrated a positive effect on the amount of trace elements in the derived breast muscles [44]. Greater levels of Fe, Zn and Se in muscles from heavy birds could reflect the increasing physiological demand of such metals with advancing age of chickens, as they are notoriously involved in the regulation of enzymatic abilities, redox reactions, and many other biochemical functions. Accordingly, previous studies already demonstrated that an improved growth performance, a better health status, and, not least, high quality meat products were achieved by higher intakes of trace elements through diet [84]. Conversely, deficiencies of these elements typically led to poor growth performance and reduced meat quality, especially in terms of oxidative stability and technological properties [85,86]. The higher level of Se in experimental products represented an obvious consequence of the targeted feed fortification with Se which, in turn, affected the retention of other inorganic elements in the muscle tissue. This was confirmed not only by the significant relationships between elements, such as Fe and Mn, and the diet treatment (Table 6), but also by previous studies already pointing out that Se influences the metabolism of other elements, such as Zn, Fe and Mn, the type of interaction (i.e., antagonistic or agonistic) being strictly related to the type of Se source [83,87,88]. However, the higher amounts of Fe, Mn and Zn observed in functional meat than the control counterpart may be explained also by a decreased excretion and a subsequent greater accumulation of these essential trace metals in tissues, as an unavoidable metabolic response of the broiler to the diet enriched with Se, at the expense of the other trace elements (see Table 2) [82,86].

The level of potentially toxic trace elements, such as As, Cd and Pb, generally showed an increasing trend with the slaughtering weight of broilers. Cd, for example, became higher in breast meats from light to heavy broilers (respectively, 0.92–1.65 µg/100g, *p* > 0.05), whereas Pb showed a perfectly opposite trend (1.12–0.54 µg/100g, *p* > 0.05). Additionally, both Cd and Pb resulted in greater accumulation in enriched than standard muscles (Cd: 0.98 µg/100g vs. 1.55 µg/100g; Pb: 0.49 µg/100g vs. 1.30 µg/100g) (Table 6). According to the statistical model, Cd was directly related to all the variation sources (*p* < 0.05), while Pb was indirectly and directly related to the weight of broilers and the dietary treatment (*p* < 0.05), respectively.

Toxic elements in broiler products originate from the feed, water, litter, and, not least, the surrounding environment and can be transmitted through the food chain, thus having a detrimental impact on the health of both animals and humans [89]. However, according to the Regulation (EC) No. 1881/2006 and subsequent amendments [90], all meat samples fell within the safety limits fixed for Cd and Pb (0.05 mg/Kg and 0.10 mg/Kg, respectively), thus not posing a threat for the human consumer.

To the best knowledge of the authors, no study has ever investigated the heavy metal contents in chickens from different market classes. Overall, our findings agreed with all those previous works pointing out higher quantities of heavy metals with the increasing age of various meat animals [91,92,93]. Additionally, the enrichment of feed with Se demonstrated an impact on the content of Cd and Pb, positively and negatively, respectively, in pectoral muscles. In this respect, the complex interactions of Se-Cd and Se-Pb in muscle, liver and pancreas of broilers were highlighted. However, the literature mainly argued peculiar antagonistic interactions of Se with Pb and Cd, which would exert a protective effect against toxicity in these organs [94,95,96].

### 3.3. Vitamin E

The content of vitamin E in control and experimental pectoral muscles of broilers from various market classes is shown in Table 7.

In breast samples, the content of the vitamin E became higher in relation to the slaughtering weight of broilers (1.57–2.20 mg/100g, *p* > 0.05) and dietary manipulation (1.23 mg/100g vs. 2.36 mg/100g, *p* > 0.05). The statistical model reported significant relation of this vitamin with the market class and the market class × dietary treatment interaction (*p* < 0.05), but not the dietary treatment (Table 7). As a result, similarly to the other nutrients, the highest content of vitamin E would be present in enriched breasts from heavy chickens.

The literature reported that the vitamin E content in poultry meat increased linearly as the dietary vitamin E supplementation increased, both in level and administration time [97,98]. However, the increased vitamin E content of functional samples was probably due also to the supplementation of Se in the diet, although the extent of the increase in vitamin E would be strictly dependent on the Se dietary source [99]. The synergic mechanism between the two nutrients has not yet been fully ascertained, but it may involve the glutathione peroxidase, an enzyme playing an important role in the detoxification of lipidic hydroperoxide. This selenoprotein is present in nearly all broiler tissues, including the gastrointestinal mucosa, where it may offer a primary barrier against the absorption of ingested hydroperoxides, thus enhancing the action of vitamin E by decreasing its oxidation extent [99], as well as in pancreas and liver, where it notoriously preserves the organ integrity and functionality, thus indirectly allowing a normal fat digestion and vitamin E absorption [100]. 

### 3.4. Nutritional Value of Conventional and Functional Meats

The consumption of functional breast meat can effectively contribute to the coverage of daily requirements of EPA + DHA, inorganic elements, and vitamin E, as established by the Italian Society of Human Nutrition (SINU) with the Dietary Reference Values of Nutrients and Energy for Italian population (LARN) [101].

According to the data reported in Table 6, the effect of feeding manipulation on breast meats was profitable, as functional products were better able than conventional products to cover the daily requirements of all the investigated nutrients. Additionally, in accordance with the effects described here, the consumption of enriched meat from heavy broilers would be preferred, as they may supply the highest intake of nutrients, when considering the nutritional requirements of an adult male (30–59 years old).

These products, for example, may guarantee a contribution of 28.84% to the daily intake of EPA ± DHA.

They also may provide a coverage of the daily intake of Mg up to 20.00%, Fe and Se up to 35.61% and 62.85%, and vitamin E up to 24% (Table 8).

## 4. Conclusions

In the production system of broiler chickens in Italy, both the market class and the dietary manipulation by supplementation of extruded FSM, Se and vitamin E are two relevant factors to consider for producing meat with higher nutritional and functional value. In the breast meat, as expected, the dietary treatment was more significant than slaughtering weight in causing the increase in n-3 PUFAs and advantageous metabolic and functional indices in enriched products. However, all the health indices were also characterized by a significant relationship with the market class × dietary treatment interaction, and therefore enriched products from heavier broilers had better functional traits than the conventional ones. On the other hand, the market class affected to a greater extent the mineral profile and content of vitamin E of breast muscles and established in most cases significant relationships of interdependence with both factors. In particular, thanks to the strong effect of the market class × dietary treatment interactions, enriched breast meat from heavier broilers were again characterized by higher contents of minerals, trace essential elements, and vitamin E and, thus, a higher nutritional value. These results lay the foundations for further research on the technological and sensorial properties of standard and enriched breasts from broilers of different market categories.

## Figures and Tables

**Table 1:** Experimental plan of the study. In each of the 72 flocks, *n* = 500 birds were allocated, and *n* = 60 breast samples were collected for a given market class intended for a given feeding program. In every case, the total of samples was split in three replicates, each consisting of *n* = 20 units.

Feeding Program		Market Class			Total (According to the Diet Program)
		Light Broiler	Medium Broiler	Heavy Broiler	
**Control**	*n* flocks	12	12	12	36
	*n* samples	60	60	60	180
	*n* replicates	3	3	3	9
**Functional**	*n* flocks	12	12	12	36
	*n* samples	60	60	60	180
	*n* replicates	3	3	3	9
**Total** **(according to the market class)**		24	24	24	**72**
		120	120	120	**360**
		6	6	6	**18**

**Table 2:** Ingredients (%) and proximate composition (%) of control and functional diet. AME = Apparent Metabolizable Energy.

		Starter	Grower 1	Grower 2	Finisher
		*Ingredients (%)*			
Corn	Control	45.70	48.53	55.27	56.88
	Functional	46.45	51.38	55.56	57.69
Soybean meal	Control	36.93	32.10	27.70	25.92
	Functional	36.40	32.22	27.73	25.65
Wheat middlings	Control	5.90	8.35	6.00	6.08
	Functional	4.00	4.00	4.00	4.00
Sunflower meal	Control	1.25	1.25	1.90	2.00
	Functional	1.00	1.00	1.00	1.00
Oil, fat	Control	4.20	5.15	5.70	5.95
	Functional	3.50	4.30	5.03	5.20
Corn gluten meal	Control	1.25	0.70	-	-
	Functional	1.32	0.55	-	-
Extruded linseed meal	Control	-	-	-	-
	Functional	2.50	2.50	3.13	3.13
Vitamin E (100,000 UI/Kg feed)	Control	-	-	-	-
	Functional	0.22	0.23	0.23	0.23
Selenium (2000 mg/Kg feed)	Control	-	-	-	-
	Functional	0.01	0.01	0.01	0.01
Premix (Min. + Vit. + Enz. + Amin.)	Control	4.77	3.92	3.43	3.17
	Functional	4.60	3.81	3.33	3.09
		***Proximate composition (%)***			
Volume	Control	100	100	100	100
	Functional	100	100	100	100
AME (Kcal/Kg)	Control	2996	3094	3187	3214
	Functional	2991	3095	3184	3215
Dry matter	Control	89.34	89.20	89.00	89.00
	Functional	89.30	89.20	89.00	89.00
Crude Protein	Control	23.32	21.30	19.10	18.40
	Functional	23.30	21.30	19.20	18.40
Crude Fiber	Control	3.12	3.10	3.00	2.90
	Functional	3.20	3.10	2.90	2.90
Ether extract	Control	6.75	7.80	8.40	8.70
	Functional	6.90	7.70	8.70	8.90
Ash	Control	6.77	5.90	5.40	5.20
	Functional	6.80	6.10	5.50	5.30
Lysine (digest)	Control	1.28	1.15	1.02	0.96
	Functional	1.28	1.15	1.02	0.96
Metionine + Cysteine (digest)	Control	0.95	0.87	0.80	0.75
	Functional	0.95	0.87	0.80	0.75
Threonine (digest)	Control	0.87	0.78	0.69	0.64
	Functional	0.87	0.78	0.69	0.64
Calcium	Control	1.00	0.90	0.80	0.70
	Functional	1.00	0.90	0.80	0.70
Phosphorus	Control	0.70	0.60	0.50	0.50
	Functional	0.70	0.60	0.50	0.50
Selenium (mg/Kg feed) *	Control	0.10	0.10	0.10	0.10
	Functional	0.40	0.40	0.40	0.40
Vitamin E (mg/Kg feed)	Control	84	70	70	70
	Functional	299	300	300	300

* Selenium: 67% sodium selenite + 33% selenomethionine.

**Table 3:** Total fat (g/100 of fresh breast meat) and fatty acid composition (g/100 of total fat) of the breast fillet of broiler according to the market class and the dietary treatment. For every market class, data are reported in terms of mean ± standard deviation of *n* = 120 representative breasts from broilers intended for conventional (*n* = 60) and supplemented (*n* = 60) feeding. For the dietary treatment, data are expressed as mean ± standard deviation of *n* = 180 representative breast muscles from light (*n* = 60), medium (*n* = 60) and heavy (*n* = 60) broilers.

%	Market Class			Dietary Treatment		Source of Variation		
	Light Broiler	Medium Broiler	Heavy Broiler	Standard	Enriched	Market Class	Dietary Treatment	Market Class × Dietary Treatment
**Total fat**	**1.68 ± 0.17 ^a^**	**2.91 ± 0.19 ^b^**	**4.03 ± 0.68 ^c^**	**3.13 ± 0.78**	**2.62 ± 0.65**	*	*	*
C14:0	0.84 ± 0.03 ^a^	0.70 ± 0.04 ^b^	0.59 ± 0.06 ^c^	0.74 ± 0.10	0.68 ± 0.12	*	NS	*
C16:0	26.91 ± 2.18	26.50 ± 2.74	25.45 ± 1.77	28.33 ± 1.05	24.25 ± 0.93	NS	*	*
C18:0	8.55 ± 0.52	8.51 ± 0.51	8.11 ± 0.32	8.57 ± 0.57	8.21 ± 0.32	NS	NS	NS
C20:0	0.17 ± 0.07	0.22 ± 0.04	0.21 ± 0.08	0.15 ± 0.04	0.26 ± 0.04	*	*	NS
**SFA**	**36.48 ± 2.36**	**35.93 ± 0.51**	**34.35 ± 1.83**	**37.78 ± 1.40**	**33.39 ± 1.08**	**NS**	*	*
C16:1n-7	3.66 ± 0.71	3.66 ± 0.62	4.07 ± 0.53	4.26 ± 0.42	3.33 ± 0.44	NS	NS	NS
C18:1n-7	31.30 ± 0.77	31.40 ± 0.35	32.02 ± 0.62	31.40 ± 0.84	31.70 ± 0.88	NS	NS	NS
**MUFA**	**34.96 ± 1.28**	**35.00 ± 1.60**	**36.09 ± 1.63**	**35.66 ± 0.95**	**35.04 ± 1.01**	**NS**	**NS**	**NS**
C18:2n-6	19.79 ± 2.89	19.16 ± 1.19	18.26 ± 1.76	20.08 ± 1.08	18.06 ± 1.64	NS	NS	*
C18:3n-6	0.52 ± 0.40	0.51 ± 0.39	0.60 ± 0.50	0.13 ± 0.03	0.95 ± 0.11	NS	NS	NS
C18:3n-3	4.90 ± 3.14 ^a^	4.96 ± 2.90 ^a^	6.54 ± 2.54 ^a^	2.77 ± 1.09	8.18 ± 0.70	*	*	*
C20:2n-6	0.28 ± 0.04	0.36 ± 0.10	0.31 ± 0.09	0.38 ± 0.07	0.25 ± 0.04	NS	*	NS
C20:4n-6	0.62 ± 0.19	0.65 ± 0.20	0.80 ± 0.27	0.89 ± 0.13	0.49 ± 0.05	NS	*	NS
C20:5n-3	0.30 ± 0.24	0.29 ± 0.23	0.44 ± 0.29	0.11 ± 0.05	0.58 ± 0.12	NS	*	NS
C22:6n-3	0.44 ± 0.24	0.44 ± 0.26	0.50 ± 0.27	0.23 ± 0.03	0.69 ± 0.15	NS	*	NS
**PUFA**	**26.90 ± 4.73 ^a^**	**26.33 ± 2.94 ^a^**	**27.44 ± 2.29 ^a^**	**24.18 ± 2.35**	**29.60 ± 1.66**	*	*	*

a, b, c in the same row indicate significantly different values among breast muscles from different market classes (*p* < 0.05, by post hoc Tukey’s HSD test); * Statistically significant (*p* < 0.05) and NS = non-significant by multiple linear model.

**Table 4:** Fatty acid metabolism indices in breast fillet of broiler according to the market class and the dietary treatment. For every market class, data are reported in terms of mean ± standard deviation of *n* = 120 representative breasts from broilers intended for conventional (*n* = 60) and supplemented (*n* = 60) feeding. For the dietary treatment, data are expressed as mean ± standard deviation of *n* = 180 representative breasts from light (*n* = 60), medium (*n* = 60) and heavy (*n* = 60) broilers. Δ^9^-DI_C16_ and Δ^9^-DI_C18_: Δ^9^-desaturase indices for C16 and C18 FAs; total Δ^9^-DI: total Δ^9^-desaturase index; Δ^5^ + Δ^6^-DI: Δ^5^ + Δ^6^-desaturase index.

Metabolic Indices	Market Class			Dietary Treatment		Source of Variation		
	Light Broiler	Medium Broiler	Heavy Broiler	Standard	Enriched	Market Class	Dietary Treatment	Market Class × Dietary Treatment
Thioesterase index	32.12 ± 1.94 ^a^	37.58 ± 2.40 ^b^	43.27 ± 2.32 ^c^	38.58 ± 4.30	36.73 ± 5.76	*	NS	NS
Elongase index	0.32 ± 0.03	0.32 ± 0.03	0.32 ± 0.02	0.30 ± 0.02	0.34 ± 0.02	NS	NS	NS
$\Delta^{9}{-\;\mathrm{DI}}_{C16\;}$	11.98 ± 1.35	12.11 ± 1.54	13.75 ± 1.09	13.09 ± 1.36	12.07 ± 1.59	NS	NS	NS
$\Delta^{9}{-\;\mathrm{DI}}_{C18\;}$	78.52 ± 1.41	78.66 ± 1.11	79.80 ± 0.73	78.57 ± 1.42	79.42 ± 0.84	NS	*	NS
${\mathrm{Total}\;\Delta }^{9}{-\mathrm{DI}}_{\;}^{\;}$	39.45 ± 1.82	39.91 ± 2.29	41.55 ± 1.76	38.65 ± 1.44	41.96 ± 1.48	NS	NS	*
$\Delta^{5}+\Delta^{6}-\mathrm{DI}$	6.10 ± 1.74	6.60 ± 1.60	7.50 ± 2.36	5.03 ± 0.54	8.44 ± 1.22	NS	*	*

a, b, c in the same row indicate significantly different values among breast muscles from different market classes (*p* < 0.05, by post hoc Tukey’s HSD test); * Statistically significant (*p* < 0.05) and NS = non-significant by multiple linear model.

**Table 5:** Functional indices of breast meat according to the market class and the dietary treatment of broilers. For each market class, data are reported in terms of mean ± standard deviation of *n* = 120 representative breast muscles from broilers intended for conventional (*n* = 60) and supplemented (*n* = 60) feeding. For the dietary treatment, data are expressed as mean ± standard deviation of *n* = 180 representative breast muscles from light (*n* = 60), medium (*n* = 60) and heavy (*n* = 60) broilers S/P: saturation index; h/H: hypocholesterolemic and hypercholesterolemic FA ratio; AI: atherogenicity index; TI: thrombogenicity index.

Functional Indices	Market Class			Dietary Treatment		Source of Variation		
	Light Broiler	Medium Broiler	Heavy Broiler	Standard	Enriched	Market Class	Dietary Treatment	Market Class × Dietary Treatment
S/P	0.59 ± 0.08	0.59 ± 0.07	0.54 ± 0.05	0.63 ± 0.04	0.52 ± 0.03	NS	*	*
h/H	2.04 ± 0.29	2.06 ± 0.29	2.19 ± 0.20	1.87 ± 0.13	2.33 ± 0.13	NS	*	*
AI	1.77 ± 0.25	1.75 ± 0.25	1.62 ± 0.17	1.91 ± 0.13	1.51 ± 0.09	NS	*	*
TI	0.85 ± 0.26	0.84 ± 0.25	0.69 ± 0.16	1.00 ± 0.13	0.58 ± 0.04	NS	*	*
n-6/n-3	5.83 ± 3.74	5.42 ± 3.29	3.25 ± 1.57	7.52 ± 2.24	2.15 ± 0.25	NS	*	*

* Statistically significant (*p* < 0.05) and NS = non-significant by multiple linear model.

**Table 6:** Contents of minerals (mg/100g, fw), trace essential elements (mg/100g and µg/100g, fw) and potentially toxic elements (µg/100g, fw) in breast muscles according to the market class and the dietary treatment of broilers. For each market class, data are reported in terms of mean ± standard deviation of *n* = 120 representative breasts from broilers intended for conventional (*n* = 60) and supplemented (*n* = 60) feeding. For the dietary treatment, data are expressed as mean ± standard deviation of *n* = 180 representative breast muscles from light (*n* = 60), medium (*n* = 60) and heavy (*n* = 60) broilers. LOD (limit of detection) of As: 0.015 µg/100g.

Analyte	Market Class			Dietary Treatment		Source of Variation		
	Light Broiler	Medium Broiler	Heavy Broiler	Standard	Enriched			
mg/100g						Market Class	Dietary Treatment	Market Class × Dietary Treatment
Na	14.93 ± 8.94	15.88 ± 10.08	27.44 ± 22.05	6.52 ± 0.43	32.32 ± 12.34	*	NS	NS
Mg	245.10 ± 179.27	250.01 ± 196.40	328.46 ± 280.02	66.84 ± 5.96	482.20 ± 91.99	*	NS	*
K	424.29 ± 406.09	411.27 ± 396.41	473.36 ± 390.72	40.21 ± 9.21	799.06 ± 49.22	*	NS	NS
Fe	1.36 ± 0.88	1.49 ± 1.09	2.21 ± 1.46	0.62 ± 0.19	2.75 ± 0.72	NS	*	NS
Zn	0.27 ± 0.17	0.40 ± 0.20	0.60 ± 0.15	0.31 ± 0.23	0.54 ± 0.11	NS	NS	*
**µg/100g**								
Mn	22.48 ± 16.25	26.92 ± 21.73	31.19 ± 28.98	6.33 ± 4.56	48.06 ± 9.89	*	*	NS
Se	8.35 ± 3.64	10.34 ± 6.19	20.11 ± 17.84	5.33 ± 1.11	20.53 ± 13.10	*	*	*
As	<LOD	0.10 ± 0.00	0.40 ± 0.25	0.18 ± 0.25	0.18 ± 0.15	NS	NS	NS
Cd	0.92 ± 0.47	1.22 ± 0.52	1.65 ± 0.44	0.98 ± 0.47	1.55 ± 0.48	*	*	*
Pb	1.12 ± 0.73	1.03 ± 0.66	0.54 ± 0.27	0.49 ± 0.18	1.30 ± 0.66	*	*	NS

* Statistically significant (*p* < 0.05) and NS = non-significant by multiple linear model.

**Table 7:** Level of vitamin E (mg/100g, fw), in breast meat according to the market class and the dietary treatment of broilers. For each market class, data are reported in terms of mean ± standard deviation of *n* = 120 representative breast muscles from broilers intended for conventional (*n* = 60) and supplemented (*n* = 60) feeding. For the dietary treatment, data are expressed as mean ± standard deviation of *n* = 180 representative breasts from light (*n* = 60), medium (*n* = 60) and heavy (*n* = 60) broilers.

Analyte	Market Class			Dietary Treatment		Source of Variation		
	Light Broiler	Medium Broiler	Heavy Broiler	Standard	Experimental	Market Class	Dietary Treatment	Market Class × Dietary Treatment
Vitamin E	1.57 ± 0.42	1.61 ± 0.45	2.20 ± 1.04	1.23 ± 0.55	2.36 ± 0.65	*	NS	*

* Statistically significant (*p* < 0.05) and NS = non-significant by multiple linear model.

**Table 8:** Coverage (%) of adequate intake (AI), and population reference intake (PRI) of certain nutrients derived from the daily consumption of control and functional breast meats from chickens of various commercial categories. Data were calculated with respect to the nutritional requirements of an adult male (30–59 y/o).

Nutrient and Relative AI or PRI			Light Broiler	Medium Broiler	Heavy Broiler
			*Coverage (%)*		
EPA + DHA	AI: 250 mg/die	Control	0.89	3.64	5.52
		Functional	3.59	13.08	28.84
Mg	PRI:240 mg/die	Control	2.71	2.61	2.90
		Functional	9.80	10.61	20.03
K	AI:39 g/die	Control	0.20	0.20	0.22
		Functional	1.12	1.21	1.51
Fe	PRI:10 mg/die	Control	5.22	4.81	8.52
		Functional	22.0	24.92	35.61
Zn	AI:2.7 mg/die	Control	7.04	8.25	14.11
		Functional	15.92	17.8	24.46
Mn	PRI:12 mg/die	Control	0.10	0.03	0.03
		Functional	0.35	0.36	0.48
Se	PRI: 55µg/die	Control	9.40	9.40	10.32
		Functional	20.92	28.25	62.85
Vitamin E	AI: 13 mg/die	Control	13.84	9.46	9.85
		Functional	15.00	15.39	24.08
